# Exploring the Role of Attentional Reorienting in the Reactive Effects of Judgments of Learning on Memory Performance

**DOI:** 10.3390/jintelligence11080164

**Published:** 2023-08-15

**Authors:** Michelle L. Rivers, Jessica L. Janes, John Dunlosky, Amber E. Witherby, Sarah K. Tauber

**Affiliations:** 1 Department of Psychology, Texas Christian University, Fort Worth, TX 76109, USA; 2 Department of Psychological Sciences, Kent State University, Kent, OH 44240, USA; 3 Department of Psychological Sciences, Creighton University, Omaha, NE 68178, USA

**Keywords:** metacognition, measurement reactivity, judgments of learning, metamemory, monitoring

## Abstract

Making judgments of learning (JOLs) while studying related word pairs can enhance performance on tests that rely on cue-target associations (e.g., cued recall) compared to studying alone. One possible explanation for this positive JOL reactivity effect is that the prompt to make JOLs, which typically occurs halfway through the presentation of each pair, may encourage learners to devote more attention to the pair during the second half of the encoding episode, which may contribute to enhanced recall performance. To investigate this idea, an online sample of participants (Experiment 1) and undergraduate students (Experiment 2) studied a set of moderately related word pairs (e.g., *dairy*–*cow*) in preparation for a cued recall test. Some participants made JOLs for each pair halfway through the presentation, whereas other participants did not. Also, some participants were presented with a fixation point halfway through the presentation, whereas other participants were not. The goal of this fixation point was to simulate the possible “reorienting” effect of a JOL prompt halfway through each encoding episode. In both an unsupervised online context and a supervised laboratory context, cued recall performance was higher for participants who made JOLs compared to those who did not make JOLs. However, presenting a fixation point halfway through the presentation of each pair did not lead to reactive effects on memory. Thus, JOLs are more effective than a manipulation that reoriented participants to the word pairs in another way (i.e., via a fixation point), which provides some initial evidence that positive reactivity for related pairs is not solely driven by attentional reorienting during encoding.

## 1. Introduction

Metamemory researchers often use *judgments of learning* (JOLs) to investigate learners’ abilities to predict their future memory performance (Rhodes 2016). These judgments—either when made immediately after study, or at a delay—can directly influence the representations of the material being judged (for reviews, see Double and Birney 2019; Double et al. 2018; Rhodes and Tauber 2011). In the current investigation, we focus on the reactive effect of making immediate JOLs on memory performance. Such *JOL reactivity* has been investigated for a variety of materials, including single words (e.g., Begg et al. 1989; Halamish 2018; Li et al. 2022; Senkova and Otani 2021; Tauber and Rhodes 2012; Tekin and Roediger 2020; Yang et al. 2015; Zechmeister and Shaughnessy 1980; Zhao et al. 2022), word pairs (e.g., Arbuckle and Cuddy 1969; Chang and Brainerd 2023; DeYoung and Serra 2021; Dougherty et al. 2005, 2018; Halamish and Undorf 2022; Janes et al. 2018; Kelemen and Weaver 1997; Maxwell and Huff 2022a, 2022b; Mitchum et al. 2016; Myers et al. 2020; Rivers et al. 2021, 2023; Soderstrom et al. 2015; Tauber and Witherby 2019; Witherby and Tauber 2017; Zhao et al. 2023), pictures (e.g., Shi et al. 2022; Sommer et al. 1995), general knowledge facts (e.g., Schäfer and Undorf 2023), and educational texts (e.g., Ariel et al. 2021; Dobson et al. 2019; Ha and Lee 2023). This research has typically revealed a memory benefit (i.e., positive reactivity) for cued recall of pairs with a semantic relationship (e.g., *coat*–*jacket*), positive reactivity for recognition of single words or pictures, no recall benefit for cued recall of unrelated word pairs (e.g., *dog*–*spoon*), and mixed results for educational material. We aimed to better understand the processes that explain the beneficial effect of making immediate JOLs on memory for related word pairs.

Why does making JOLs enhance memory? One possibility, proposed by Dougherty et al. (2005), is that “making a metacognitive judgment forces participants to process the to-be-remembered item more thoroughly than they would if no judgment was made” (p. 1110; but see Dougherty et al. 2018). Along these same lines, Zhao et al. (2022) proposed the *enhanced learning engagement* theory, which posits that positive reactivity results from the enhanced engagement that results from the requirement to make JOLs during encoding (for similar ideas, see Mitchum et al. 2016; Murphy et al. 2023; Rivers et al. 2021; Shi et al. 2022; Tauber and Witherby 2019). Such enhanced processing could involve a variety of factors (e.g., attention, effort), none of which are mutually exclusive. For example, making JOLs could lead to learners engaging in more effective or elaborative encoding strategies (Sahakyan et al. 2004; Tekin and Roediger 2020; but see Mitchum et al. 2016; Rivers et al. 2021). Or, perhaps making JOLs results in a strengthening of the information used to make judgments (such as the relatedness of words within a word pair), which leads to benefits on criterion tests sensitive to such cues (e.g., Myers et al. 2020; Soderstrom et al. 2015). Finally, JOLs may promote positive reactivity by reducing mind wandering during encoding. Consistent with this idea, memory researchers have solicited JOLs as a means to ensure participants pay attention during encoding (e.g., Carpenter and Schacter 2018). In the current investigation, we were interested in the role of attention in explaining positive reactivity, and this is our focus for the remainder of the Introduction. We return to other explanations in the Discussion.

As a direct investigation into the role of attention in explaining positive reactivity, Shi et al. (2022, Experiment 3) investigated the degree to which participants’ mind wandering differed based on whether they made JOLs while learning pictures. Undergraduate students learned four blocks of scene pictures (e.g., an airport), and pictures were presented for 6 s each. For two of the blocks, students made JOLs (on a 0–100 slider scale) during the 6 s encoding window, whereas for the other two blocks, students did not make JOLs. During each block, two mind-wandering probes appeared at a random point during encoding that asked students to rate the extent to which they were concentrating on the task on a 1–7 scale (1 = *I was fully concentrating on the task*; 7 = *I was fully mind-wandering*). Following the four learning blocks and a brief distractor task, students completed an old-new recognition test for the pictures they learned. Positive reactivity was observed for pictures that were judged, and reports of mind wandering were lower in the JOL conditions compared to the no-JOL conditions. Furthermore, the difference in mind wandering ratings between the JOL and no-JOL conditions predicted the positive reactivity effect, and the reactivity effect was partially mediated by reduced self-reported mind wandering. In a follow-up experiment (Experiment 4), instructions intended to increase learners’ motivation—and presumably increase engagement for non-judged items—reduced the positive reactivity effect. Thus, Shi et al. found initial evidence that JOLs facilitate memory for pictures through enhanced attention (i.e., reduced mind wandering during encoding).

In the investigation by Shi et al., learners were given the full encoding period (i.e., 6 s) to make their JOLs for each picture. However, many investigations soliciting JOLs during encoding have used a slightly different procedure. Consider the procedure used by Soderstrom et al. (2015) in one of the first investigations to explore the mechanisms of JOL reactivity (and adopted in many investigations since). Participants learned a series of word pairs and either made or did not make JOLs. For the no-JOL groups, word pairs were presented for 8 s each, and the entire presentation time was used to learn each pair. Attention may have waned, and mind wandering may have increased across the encoding episode. In contrast, for the JOL group, judgments were elicited halfway through the presentation of each pair (i.e., after 4 s) while the pair remained visible. Thus, encoding time was broken up—learners first studied the pair for 4 s and were then prompted to make a JOL, which could have resulted in learners “reorienting” to the pair. That is, the prompt to make JOLs may have encouraged learners to devote more attention to the pair during the second half of the encoding episode, leading to benefits on a later memory test. This “attentional reorienting” hypothesis was first proposed by Tauber and Witherby (2019) as a potential explanation for positive reactivity for related word pairs (observed in younger but not older adults) and was partially informed by prior research suggesting that attentional refreshing during working memory span tasks predicts episodic memory (e.g., Loaiza and McCabe 2012). However, they did not evaluate the role of attentional reorienting in JOL reactivity.

In the current experiments, we investigated whether attentional reorienting during encoding contributes to positive reactivity for related word pairs. We used related word pairs as stimuli because they consistently show a positive reactivity effect, at least with younger adult participants (e.g., Halamish and Undorf 2022; Janes et al. 2018; Maxwell and Huff 2022a, 2022b; Myers et al. 2020; Rivers et al. 2021; Soderstrom et al. 2015; Tauber and Witherby 2019; Witherby and Tauber 2017; but see DeYoung and Serra 2021; Mitchum et al. 2016). Participants (an online sample in Experiment 1 and undergraduates in Experiment 2) learned these related word pairs and either made or did not make JOLs halfway through encoding each pair. We included an additional manipulation during encoding; that is, halfway through the presentation of each pair, participants were briefly presented with a fixation point (“+”). Researchers often use external cues (e.g., arrows, fixation points, dots) to direct participants’ attention to presented stimuli (e.g., Posner 1980). The goal of this fixation point was to simulate the “reorienting” effect of a JOL prompt halfway through each encoding episode. Following encoding, all participants completed a cued recall test on the pairs they learned. Based on prior research with related word pairs, we predicted a positive reactivity effect; that is, recall performance would be higher for the JOL group than the no-JOL group. And, if attentional reorienting contributes to positive reactivity, participants who receive a fixation point halfway through the presentation should also show positive reactivity relative to those who do not make JOLs. We also included a post-experiment questionnaire to better understand how learners made their judgments.

## 2. Experiment 1

### 2.1. Methods

#### 2.1.1. Participants

We used a rule-of-thumb of 45 participants per group (180 participants total). A power analysis conducted using G*Power 3.1.9.7 (Faul et al. 2007) for an independent-samples *t*-test (e.g., between the JOL and no-JOL groups) with power set at 0.80 and two-tailed α = 0.05 indicated that this sample size provided sufficient sensitivity to detect an effect of Cohen’s *d* = 0.60 or higher.

We posted timeslots exceeding our target sample size on Amazon’s Mechanical Turk and provided compensation of USD 0.50 per participant. All participants were from the United States, fluent in English, and had an approval rate of 95% or higher on the platform. Data were analyzed from the 216 participants (*M*_age_ = 37.3 years, range 18–84 years; 62% female; 75% White, 7% Black/African American, 7% Asian, 6% Latino/Hispanic, 3% mixed race/ethnicity, 1% Native American) who completed the experiment without technical issues and submitted a valid completion code. An additional 12 participants completed the experiment but were removed from the analysis (4 from the JOL + fixation group, 6 from the JOL + no-fixation group, and 2 in the no-JOL + fixation group); 9 participants reported cheating, and 3 participants did not attempt recall when prompted.

#### 2.1.2. Design

Experiment 1 used a 2 (judgment group: JOL, no-JOL) × 2 (fixation point: with, without) between participant design. Participants were randomly assigned by Qualtrics software to the JOL + fixation group (*n* = 53), the JOL + no-fixation group (*n* = 51), the no-JOL + fixation group (*n* = 47), and the no-JOL + no-fixation group (*n* = 65).

#### 2.1.3. Materials

Materials were 60 weakly related paired associates (e.g., *dairy*–*cow*; average relatedness = 0.15, *SD* = 0.03). Pairs were modified from (Tauber and Witherby 2019, Experiment 3; created from the Nelson et al. 2004, norms). Cue and target words did not differ in length, *t*(59) 1.43, *p* = 0.16, frequency, *t*(59) = 1.93, *p* = 0.06, or number of syllables (*t* < 1). Experiments were programmed using Qualtrics software.

#### 2.1.4. Procedure

Participants were instructed that they would be learning a series of word pairs for an upcoming cued recall test (i.e., “Please do your best to remember each pair, so that when you are tested later you will be able to recall the second word when shown the first word of each pair.”) Pairs were presented individually for 8 s each.

**Figure 1:**
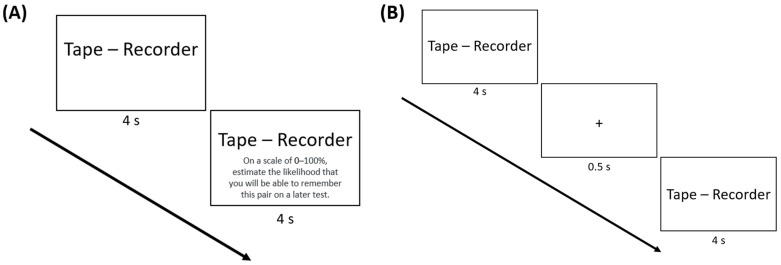
Procedure for the (**A**) JOL and no-fixation group and (**B**) no-JOL and fixation group of Experiment 1. Participants in the JOL groups were prompted to make a judgment of learning halfway through the presentation of each word pair while the pair remained on screen. Participants in the fixation groups were presented with a fixation point (“+”) for 500 ms halfway through the presentation of each pair. For the JOL groups, this fixation point appeared right before the JOL prompt.

In the JOL groups, participants were prompted to type a JOL into a text box halfway through the presentation of each pair (i.e., indicate the likelihood of remembering the pair on a later test on a scale from 0 to 100) while the pair remained on screen. Participants had the remaining 4 s to make their JOL; see Figure 1A. However, if participants failed to make a JOL in the remaining 4 s, they received an error message and had to enter their judgment before proceeding (while the pair remained on screen). Participants in the no-JOL groups did not make JOLs.

In the fixation groups, a fixation point (“+”) appeared in the center of the screen for 500 ms halfway through the presentation of each pair (i.e., after 4 s; Figure 1B). Participants were instructed to use this fixation point as a reminder to keep studying the pair for the remaining 4 s. For the JOL groups, this fixation point appeared right before the JOL prompt (also for 500 ms). Participants in the no-fixation groups were not presented with the fixation point during the pair presentation.

After a 3 min distractor task involving verifying whether math equations were true or false, participants engaged in a self-paced cued recall test on which they were given the cue and were asked to recall the target (e.g., dairy–?). All pairs were tested one at a time, and no feedback was provided. The order of presentation of the pairs during encoding was the same for all participants, whereas the order of presentation during cued recall was randomized anew for each participant.

Following cued recall, participants were asked two exploratory questions: (1) “So far, what do you think this experiment is about?” and (2) “Do you think this is a memory experiment?” Participants in the JOL groups were also asked, “How did you make your judgments (that is, why did you give some word pairs a higher judgment and others a lower judgment)?”

At the end of the experiment, participants were asked, “At any point in the experiment, did you cheat and write down the words you were studying? You will still receive payment if the answer is yes, so please answer honestly.” The 9 participants who responded “yes” to this question were excluded from the analysis.

### 2.2. Results

Item-level data resulting from Experiments 1 and 2 of the current investigation are available on the Open Science Framework at https://doi.org/10.17605/OSF.IO/69S8E.

All reported statistical tests are two-tailed. To supplement null-hypothesis significance testing, for *t*-tests, we also report Bayes factors. Bayes factors are the ratio of the likelihood of the data given the alternative hypothesis to the likelihood of the data given the null hypothesis (*BF*_10_). A *BF*_10_ greater than 1 suggests that the alternative hypothesis is more likely, a *BF*_10_ of 1 suggests that both hypotheses are equally likely, and a *BF*_10_ less than 1 suggests that the null hypothesis is more likely (for discussion, see Rouder et al. 2009). In cases where the null hypothesis is more likely, Bayes factors are reported as the reciprocal *BF*_01_ for ease of interpretation. Effect sizes for *t*-tests are reported in terms of Hedges’ *g* (formulas from Lakens 2013).

Cued recall responses were marked as correct if they matched the target exactly. The JOL magnitudes for both experiments are presented in Table 1.

#### 2.2.1. Recall Performance

**Figure 2:**
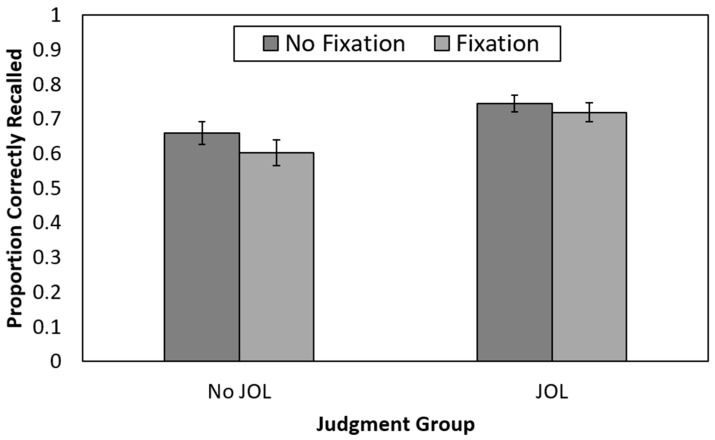
Recall performance as a function of judgment and fixation group in Experiment 1. JOL = judgment of learning. Error bars reflect the standard error of each mean.

Recall performance is presented in Figure 2. Only one participant (in the no-JOL + no-fixation group) failed to correctly recall any items. Histograms displaying the full distribution of recall performance by group (for both Experiments 1 and 2) are available on the Open Science Framework (https://osf.io/uqfph).

A 2 (judgment group: no-JOL vs. JOL) × 2 (fixation group: fixation point vs. no-fixation point) between-participants ANOVA revealed that recall was significantly higher for those who made JOLs (*M* = 0.73, *SE* = 0.02) than for those who did not make JOLs (*M* = 0.63, *SE* = 0.02), *F*(1,212) = 10.27, *p* = 0.002, η_p_^2^ = 0.05. Recall did not significantly differ for those presented with a fixation point during encoding (*M* = 0.66, *SE* = 0.02) compared to those who were not presented with a fixation point (*M* = 0.70, *SE* = 0.02), *F*(1,212) = 1.72, *p* = 0.19, η_p_^2^ = 0.008. The 2 × 2 interaction was not significant, *F*(1,212) = 0.27, *p* = 0.61, η_p_^2^ = 0.001. Thus, making JOLs benefited recall, whereas a fixation point presented halfway through each encoding episode did not.

#### 2.2.2. Post-Experiment Questionnaire Responses and Conditional Analyses

When asked what they thought the experiment was about, most participants said memory (65.3%) or word associations (9.7%). Other responses (25%) included attention/focus, metamemory, confidence, or “no idea.” When specifically asked if they thought the experiment was about memory, 193 participants (89.4%) responded yes.

Participants in the JOL groups were also asked how they made their judgments. Five participants did not respond to this question, or it was obvious from their responses that they did not understand the question (e.g., “I have no idea what this means”). For the other 99 respondents, authors M.L.R. and A.E.W. independently coded responses into three categories: “relatedness/ association,” “memory,” and “other/no idea” (e.g., serial position, familiarity, difficulty, common sense). Some responses fell into multiple categories. The two coders agreed on 94% of responses, and disagreements were resolved through discussion. Table 2 contains sample responses from each category and the frequency with which participants made each response.

Next, we compared recall performance between participants who did and did not mention using relatedness to inform their judgments. The former group of participants included those who mentioned relatedness, regardless of whether or not they also mentioned memory or something else, whereas the latter group of participants were those who did not mention relatedness at all. An independent samples *t*-test revealed that recall was significantly higher for participants who reported using relatedness (*M* = 0.77, *SE* = 0.02) compared to those who did not (*M* = 0.68, *SE* = 0.04), *t*(97) *=* 2.66, *p* = 0.009, 95% CI [.03, 0.17], *g*_s_ = 0.58, *BF*_10_ = 1.28.

## 3. Experiment 2

Given that Experiment 1 was conducted online and participants were unsupervised, it is impossible to know how engaged participants were during the learning task. In particular, the fixation point presented during encoding may have been ignored by distracted participants. If so, an in-person investigation during which participants are supervised may reveal an impact of the fixation point on later memory performance. Indeed, some studies have found differences in outcomes between investigations conducted in supervised lab contexts versus unsupervised online contexts (e.g., Simone et al. 2023). Thus, Experiment 2 was conducted in a supervised laboratory context to provide additional confidence that participants were attending to the material presented to them. Our goal was to replicate the primary outcomes of Experiment 1.

### 3.1. Methods

As in Experiment 1, we used a rule-of-thumb of 45 participants per group (135 participants total). Experiment timeslots were posted every week via the Psychology department’s SONA system at Texas Christian University. A total of 160 undergraduate students (*M*_age_ = 19.68 years, range 17–29 years; 64% female; 70% White, 14% Latino/Hispanic, 6% Black/African American, 6% Asian, 4% mixed race/ethnicity) participated in exchange for partial course credit in their Psychology course.

Participants were run in person in small groups of up to 8, and each participant was run in an individual cubicle with a computer. An undergraduate or graduate research assistant oversaw data collection and ensured participants were attending to the material on screen (e.g., not using their cell phones during the experiment). Experiment 2 used the same materials and followed the same procedure as Experiment 1, except we did not include the JOL + fixation group.

That is, participants were randomly assigned (by the Qualtrics software) to the JOL (*n* = 46), no-JOL (*n* = 58), and fixation (*n* = 56) groups. Participants in the JOL group followed the same procedure as the JOL (and no-fixation) group as Experiment 1; participants in the no-JOL group followed the same procedure as the no-JOL (and no-fixation) group as in Experiment 1; participants in the fixation group followed the same procedure as the no-JOL and fixation group as Experiment 1. None of the participants reported cheating during the experiment.

### 3.2. Results

#### 3.2.1. Recall Performance

**Figure 3:**
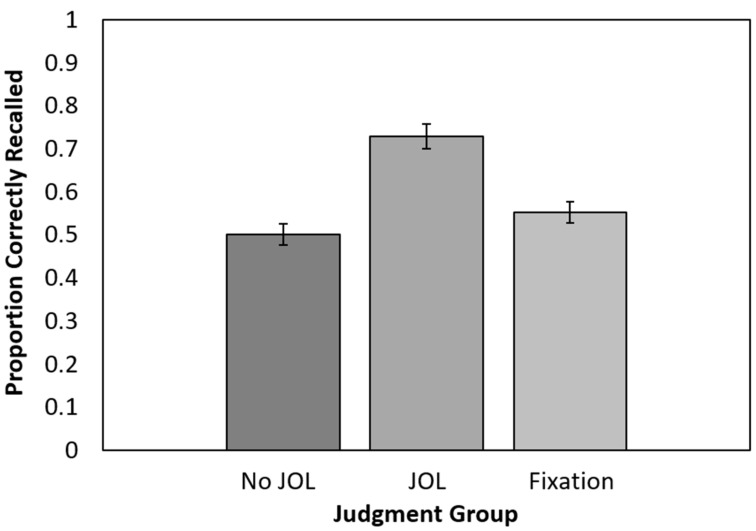
Recall performance as a function of group assignment in Experiment 2. JOL = judgment of learning. Error bars reflect the standard error of each mean.

Recall performance is presented in Figure 3. Only one participant (in the no-JOL group) failed to correctly recall any items. A one-way ANOVA conducted on cued recall performance revealed a significant effect of the JOL group, *F*(2, 157) = 19.57, *p* < 0.001, η_p_^2^ = 0.91. Follow-up *t*-tests indicated that recall was significantly higher for the JOL group than the no-JOL group, *t*(102) = 6.24, *p* < 0.001, 95% CI [0.16, 0.30], *g*_s_ = 1.22, *BF*_10_ > 100, and higher for the JOL group than the fixation point group, *t*(100) = 4.64, *p* < 0.001, 95% CI [0.10, 0.25], *g*_s_ = 0.92, *BF*_10_ > 100. Recall did not significantly differ between the fixation point and no-JOL groups, *t*(112) = 1.39, *p* = 0.17, 95% CI [−0.02, 0.12], *g*_s_ = 0.26, *BF*_01_ = 0.10. As in Experiment 1, making JOLs benefited recall, whereas a fixation point presented halfway through each encoding episode did not.

#### 3.2.2. Post-Experiment Questionnaire Responses and Conditional Analyses

When asked what they thought the experiment was about, participants said memory/metamemory (61.88%), word associations (13.1%), or other/no idea (25%). When asked if they thought the experiment was about memory, 138 participants (86.25%) responded yes.

Participants in the JOL groups were also asked how they made their judgments. One participant did not understand the question. As in Experiment 1, authors M.L.R. and A.E.W. independently coded the other responses into categories; the two coders agreed on 91% of responses, and disagreements were resolved through discussion. Table 2 contains sample responses from each category and the frequency with which participants made each response.

The JOL group most frequently reported using relatedness (as opposed to memory or some other cue) to inform their judgments. No significant difference in cued-recall performance was observed between participants who reported using relatedness to inform their judgments (*M* = 0.74, *SE* = 0.03) compared to those who did not report using relatedness (*M* = 0.69, *SE* = 0.06), *t*(44) = 0.75, *p* = 0.46, 95% CI [−0.09, 0.19], *g*_s_ = 0.27, *BF*_01_ = 0.09.

## 4. Discussion

Is positive reactivity for related word pairs driven by attentional reorienting during encoding? The outcomes of the current investigation do not support this explanation. Although we did find large, positive reactivity effects for participants who made JOLs, presenting a fixation point halfway through the presentation of each encoding episode did not lead to any memory benefits compared to studying alone. These outcomes were found in an unsupervised online context as well as in a supervised laboratory context.

Our conclusions were supported by outcomes from continuously cumulating meta-analyses (CCMA; Braver et al. 2014) across the two experiments. CCMA results for cued recall performance are reported in Table 3. Replicating prior research (e.g., Tauber and Witherby 2019; Witherby and Tauber 2017), participants who made JOLs outperformed those who did not make JOLs (pooled *g*_s_ = 0.74). Comparing effect sizes in Table 2, the JOL reactivity effect was larger with undergraduate students (in Experiment 2) compared to the online sample (in Experiment 1), and the *Q* statistic indicated significant heterogeneity between the two experiments. This difference in reactivity across experiences may be because the Experiment 1 sample consisted of some older adults, whose memory can be less influenced by making JOLs, at least for related word pairs (Tauber and Witherby 2019).

One important caveat worth mentioning is regarding our JOL reactivity outcomes. In the JOL groups, participants were presented with the JOL prompt halfway through the presentation of each word pair (i.e., after 4 s). However, if participants did not make a JOL during this time, they received an error message and were required to enter their judgment (while the pair remained on screen) before proceeding. Unfortunately, we did not collect data on how often participants failed to enter their JOLs within the allotted time (i.e., how often error messages were presented, and how long participants took to make their JOLs in such cases). Thus, the longer encoding time for the JOL groups is a potential confound of our procedure. Note that similar patterns of JOL reactivity (i.e., positive reactivity for related word pairs) have been found when using similar materials and procedures, except that the encoding time for the JOL and no-JOL groups is kept consistent (e.g., Tauber and Witherby 2019). Nevertheless, our procedure limits us from making causal claims about the impact of JOLs on cued-recall performance.

Another CCMA revealed that participants who made JOLs outperformed those who studied pairs and were presented with a fixation point halfway through the presentation (pooled *g*_s_ = 0.78). That is, contrary to the idea that attentional reorienting during encoding contributes to positive reactivity, JOLs were still more effective than a manipulation that reoriented participants in another way (i.e., via a fixation point). However, one important limitation is that we did not include a direct measure of participants’ attention. Making JOLs may re-orient participants’ attention in a way that was not mimicked by the presence of a fixation point halfway through encoding, or they may enhance global attention to judged items (Shi et al. 2022). Thus, future JOL reactivity research should include measures of attention during encoding, such as mind-wandering probes (Shi et al. 2022) or eye-tracking (Carbajal et al. 2018), to better estimate the potential role of enhanced attention and/or specific types of processing invoked by making JOLs.

Despite these important caveats, our outcomes are consistent with other evidence suggesting that the effect(s) of making JOLs on memory is not solely explained by enhanced attention during encoding. In an investigation by Dougherty et al. (2018), participants learned a series of unrelated word pairs. Some participants made JOLs during learning, whereas others did not. During a random 20% of learning trials, participants completed a dot-probe task that required them to press a button any time they saw a probe (i.e., an asterisk) appear on screen. The idea was that if participants who were required to make JOLs paid more attention to learning word pairs, they might perform worse and respond slower on the dot-probe task (compared to participants who were not required to make JOLs). Contrary to this hypothesis, performance and reaction time on the dot-probe task did not differ between participants who made JOLs and those who did not make JOLs. Although the experimental procedure and materials used by these authors differed from those used in the present study (for example, participants in the investigation by Dougherty et al. (2018) engaged in a recall attempt before making their JOLs), the conclusion is similar: any benefit resulting from making JOLs during the study is not solely due to an increase in attention during encoding.

Additionally, although we replicated the positive reactivity effect typically observed for related word pairs (Janes et al. 2018; Myers et al. 2020; Rivers et al. 2021; Soderstrom et al. 2015; Tauber and Witherby 2019; Witherby and Tauber 2017), not all research finds positive reactivity (e.g., Ariel et al. 2021; Dougherty et al. 2018; Schäfer and Undorf 2023; Tauber and Rhodes 2012). Multiple studies have found negative reactivity for unrelated word pairs (e.g., Janes et al. 2018; Mitchum et al. 2016), leading researchers to develop additional mechanisms for reactivity. One such mechanism argues that when learning difficult material, the requirement to concurrently monitor one’s learning leads to dual-task costs to memory performance (i.e., the dual-task hypothesis, Mitchum et al. 2016; see also Janes et al. 2018). Accordingly, making JOLs impairs cued recall for unrelated pairs because they are more difficult to learn than related pairs. Additionally, enhanced attention cannot provide a complete explanation for positive reactivity. Although Shi et al. (2022) found that reports of mind wandering were lower in JOL conditions compared to no-JOL conditions and positive reactivity was partially mediated by reduced mind wandering, positive reactivity survived even after controlling for the effect of making JOLs on mind wandering.

If positive reactivity for related word pairs is not driven (solely) by enhanced attention, then what else explains the effect? One prominent explanation that has received substantial support is the *cue-strengthening hypothesis* (Soderstrom et al. 2015), which states that if the cue used to inform JOLs is relevant to a criterion test, positive reactivity will occur for that material. In the case of related pairs, participants often use the relatedness of two words in a pair to inform their judgments (e.g., making higher judgments for related than unrelated word pairs, e.g., Koriat 1997). Compared to no-JOL conditions, making JOLs increases the processing of cue-target associations, which can improve performance on tests of cued recall (e.g., Halamish and Undorf 2022; Maxwell and Huff 2022a, 2022b; Myers et al. 2020; Rivers et al. 2021, 2023; Soderstrom et al. 2015). Conditional analyses based on our post-experiment questionnaire, in which participants in the JOL groups were asked how they made their judgments, provide some indirect support for this hypothesis. In particular, recall was (significantly in Experiment 1 and numerically in Experiment 2) higher for participants who mentioned using relatedness or association as a cue to inform their judgments compared to those who reported using other cues (e.g., memory, serial position, etc.). However, these outcomes should be interpreted with caution given that cell sizes were much larger for those who reported using relatedness/association compared to other cue types (see Table 2).

Finally, our outcomes (and perhaps some of our conclusions) are limited to the specific participants, materials, encoding conditions, and retrieval conditions investigated in the current experiments. Thus, future research should continue to investigate JOL reactivity with different materials (e.g., word pairs with varying degrees of relatedness, Chang and Brainerd 2023; Maxwell and Huff 2022a), method variations (e.g., self-paced versus experimenter-paced judgments, Janes et al. 2018), different types of judgments (e.g., relatedness judgments, Halamish and Undorf 2022; Maxwell and Huff 2022b), different test types (e.g., free recall or recognition; Myers et al. 2020), and other populations (e.g., older adults, Tauber and Witherby 2019) for a more complete understanding of JOL reactivity across a multitude of contexts.

## 5. Conclusions

Taken together, research on JOL reactivity has revealed that the mere act of measuring monitoring during learning can directly influence memory. Multiple mechanisms have been proposed to explain JOL reactivity effects, and some mechanisms may be more relevant in some contexts than in others. Our outcomes suggest that positive reactivity for related word pairs is not solely explained by attentional reorienting during encoding.

## Figures and Tables

**Table 1:** Mean magnitudes of judgments of learning in Experiments 1 and 2.

Experiment/Manipulation	Mean (SE)
1, JOL	65.04 (3.03)
1, JOL + Fixation	61.35 (2.80)
2, JOL	67.80 (2.93)

*Note.* JOL = judgment of learning. *SE* = standard error of the mean.

**Table 2:** Post-experiment questionnaire responses in Experiments 1 and 2.

Type of Response	Example Response	Experiment 1 Frequency (%)	Experiment 2 Frequency (%)
Relatedness/Association	“…how easily and common I thought it would be to associate the two words together.”	68 (68.7%)	37 (80.4%)
Memory	“I just thought they would be easy/hard to remember.”	14 (14.1%)	2 (4.3%)
Other/No Idea	“... I started giving myself lower judgment towards word pairs at the end rather than the beginning.”	22 (22.2%)	11 (23.9%)

*Note.* Participants who make judgments of learning were asked how they made their judgments, and responses were coded into three categories (Relatedness/Association, Memory, and Other/No Idea). Some responses fell into multiple categories.

**Table 3:** Continuously Cumulating Meta-Analysis (CCMA) outcomes for cued recall performance.

Comparison	Mean Diff	*S*_pooled_	*t*	*p* (2-Tailed)	*g*_s_	*Z*
JOL vs. No-JOL						
Expt 1	0.08	0.23	1.98	0.05	0.37	1.96
Expt 2	0.23	0.18	6.24	<0.001	1.22	5.73
CCMA Results				<0.001	0.74	5.44
JOL vs. Fixation						
Expt 1	0.14	0.22	3.25	0.002	0.65	3.15
Expt 2	0.18	0.19	4.63	<0.001	0.92	4.40
CCMA Results				<0.001	0.78	5.34

*Note.* Mean diff: mean difference in cued recall performance between (a) those who made JOLs versus those who did not (top panel: JOL vs. no-JOL) and (b) those who made JOLs versus those who were exposed to a fixation point halfway through the presentation (JOL vs. fixation). For the JOL vs. no-JOL comparison, data from Experiment 1 compares the JOL + no-fixation point group versus the no-JOL + no-fixation point group. For the JOL vs. fixation comparison, data from Experiment 1 compares the JOL + no-fixation point group and the no-JOL + no-fixation group. Expt = Experiment. JOL = judgment of learning. The effect size homogeneity test was significant for the JOL vs. no-JOL CCMA [*Q*(1) = 8.90, *p* = 0.003], but not for the JOL vs. fixation CCMA [*Q*(1) = 0.80, *p* = 0.37].

## Data Availability

The data presented in this investigation are openly available in the Open Science Framework at https://osf.io/69s8e/, DOI: 10.17605/OSF.IO/69S8E.
